# Impaired survival of regulatory T cells in pulmonary sarcoidosis

**DOI:** 10.1186/s12931-015-0265-8

**Published:** 2015-09-16

**Authors:** Caroline E. Broos, Menno van Nimwegen, Alex Kleinjan, Bregje ten Berge, Femke Muskens, Johannes C.C.M. in ’t Veen, Jouke T. Annema, Bart N. Lambrecht, Henk C. Hoogsteden, Rudi W. Hendriks, Mirjam Kool, Bernt van den Blink

**Affiliations:** Department of Pulmonary Medicine, Erasmus MC, ‘s Gravendijkwal 230, 3015 CE Rotterdam, The Netherlands; Department of Pulmonology, Sint Franciscus Gasthuis, Kleiweg 500, 3045 PM Rotterdam, The Netherlands; Department of Pulmonology, Academic Medical Centre/University of Amsterdam, Meibergdreef 9, 1105 AZ Amsterdam, The Netherlands; Laboratory of immunoregulation and mucosal immunology, VIB Inflammation Research Center, Technologiepark 927, 9052 Ghent, Belgium; Department of Respiratory Medicine, University Hospital Ghent, De Pintelaan 185, 9000 Ghent, Belgium

## Abstract

**Background:**

Impaired regulatory T cell (Treg) function is thought to contribute to ongoing inflammatory responses in sarcoidosis, but underlying mechanisms remain unclear. Moreover, it is not known if increased apoptotic susceptibility of Tregs may contribute to an impaired immunosuppressive function in sarcoidosis. Therefore, the aim of this study is to analyze proportions, phenotype, survival, and apoptotic susceptibility of Tregs in sarcoidosis.

**Methods:**

Patients with pulmonary sarcoidosis (*n* = 58) were included at time of diagnosis. Tregs were analyzed in broncho-alveolar lavage fluid and peripheral blood of patients and healthy controls (HC).

**Results:**

In sarcoidosis patients no evidence was found for a relative deficit of Tregs, neither locally nor systemically. Rather, increased proportions of circulating Tregs were observed, most prominently in patients developing chronic disease. Sarcoidosis circulating Tregs displayed adequate expression of FoxP3, CD25 and CTLA4. Remarkably, in sarcoidosis enhanced CD95 expression on circulating activated CD45RO^+^ Tregs was observed compared with HC, and proportions of these cells were significantly increased. Specifically sarcoidosis Tregs - but not Th cells - showed impaired survival compared with HC. Finally, CD95L-mediated apoptosis was enhanced in sarcoidosis Tregs.

**Conclusion:**

In untreated patients with active pulmonary sarcoidosis, Tregs show impaired survival and enhanced apoptotic susceptibility towards CD95L. Increased apoptosis likely contributes to the insufficient immunosuppressive function of sarcoidosis Tregs. Further research into this field will help determine whether improvement of Treg survival holds a promising new therapeutic approach for chronic sarcoidosis patients.

**Electronic supplementary material:**

The online version of this article (doi:10.1186/s12931-015-0265-8) contains supplementary material, which is available to authorized users.

## Background

Sarcoidosis is a multisystem granulomatous disorder of unknown cause, often affecting the lungs [1]. The disease is characterized by an exaggerated T helper (Th)1/Th17 response upon exposure to one or several antigens in genetically susceptible individuals [2, 3]. Clinical presentation and disease prognosis vary greatly. Although the majority of the patients undergo spontaneous resolution, a substantial proportion develops chronic, progressive disease with need for therapy [1]. Factors that determine granuloma fate remain to be elucidated [1, 3].

Failure of immune regulatory mechanisms to limit duration of inflammation has been suggested to contribute to persisting granulomatous responses in sarcoidosis [4], and may explain the need for immunosuppressive drugs. Effective immunosuppressive agents for (chronic) sarcoidosis include corticosteroids and anti-TNF agents [5]. Interestingly, these drugs can induce Th cell apoptosis, while sparing or even inducing regulatory T cell (Treg) proportions and function [6–10], thereby favoring an anti-inflammatory milieu.

Tregs are an indispensable subset of T cells with strong immunosuppressive capacities on a wide range of immune cells, including Th cells, B cells, and antigen presenting cells [11]. They have a fine sensitivity for immune dynamics, mediated by interleukin(IL)-2 signaling, and can quickly adjust their numbers and function during immune challenge [12]. Upon activation, Th cells produce IL-2, which contributes to Treg proliferation and survival [12]. Appropriate Treg function is required to terminate the immune response after antigen eradication, thus preventing (redundant) continuing inflammation [12]. Importantly, defective Treg function contributes to induction, sustainment or progression of various autoimmune diseases, including rheumatoid arthritis (RA) and systemic lupus erythematosus (SLE), but also granulomatous disorders such as antineutrophil cytoplasmic antibody-associated vasculitis (AAV) and hypersensitivity pneumonitis (HP) [13–15].

The role of Tregs in the pathogenesis of sarcoidosis remains controversial. An impaired immunosuppressive function of sarcoidosis peripheral blood (PB) Tregs has been reported consistently [4, 16–18]. Furthermore, broncho-alveolar lavage fluid (BALF)-derived Treg suppressive efficacy was found to increase during short-term inhaled vasoactive intestinal peptide (VIP)-treatment in pulmonary sarcoidosis, which was also associated with amelioration of clinical symptoms (i.e. dyspnea and cough) [19]. Decreased inhibition of Th cell proliferation and cytokine production by Tregs therefore likely contributes to the ongoing and exaggerated immune responses seen in sarcoidosis. Since contradictory results have been reported about Treg proportions with respect to Th cells in both BALF and PB of sarcoidosis patients (*See for review* [3]:), it remains to be determined what mechanism(s) underlies this impaired immunosuppressive function [3].

Pro- and anti-apoptotic pathways play an important role in Treg homeostasis [12]. Intriguingly, in sarcoidosis granulomas and PB increased proportions of activated (i.e. CD45RO^+^FoxP3^high^) proliferating Tregs are found [4]. The significance of this finding for sarcoidosis pathogenesis remains unclear, since CD45RO^+^FoxP3^high^ Tregs are described to be both more suppressive and more sensitive towards apoptosis in healthy individuals [20]. Although it has been suggested that increased apoptotic susceptibility of Tregs may contribute to an impaired immunosuppressive function in sarcoidosis [4], it remains unknown if survival of patient-derived Tregs is affected.

Therefore, the aim of this study is to analyze proportions, phenotype, survival, and apoptotic susceptibility of sarcoidosis Tregs. Our results demonstrate that in patients with active pulmonary sarcoidosis, Tregs show impaired survival and enhanced apoptotic susceptibility towards CD95L.

## Materials and methods

### Study design and subjects

Patients with pulmonary sarcoidosis were included at time of diagnosis. The diagnosis of sarcoidosis was made conform the guidelines of the ATS/ERS/WASOG [1].

Exclusion criteria were use of immunomodulatory medication 3 months prior to study inclusion; respiratory tract infection 4 weeks prior to study inclusion; concomitant pulmonary disease (including chronic obstructive pulmonary disorder and asthma), autoimmune diseases, malignancies, human immunodeficiency virus seropositivity, pregnancy, and allergies.

For this study, in total 58 newly diagnosed sarcoidosis patients donated PB and/or BALF. Due to limitations in the number of cells isolated per patient, we were not able to perform all experiments on all patients. Furthermore, 47 healthy controls donated PB and 5 healthy controls underwent bronchoscopy with BAL for this study.

The Medical Ethical Committee of the Erasmus MC Rotterdam approved this study. Written informed consent was obtained from every participant before study inclusion. Further subject characteristics are shown in Additional file 1: Table S1.

### Study materials

Bronchoscopy with BAL and mucosal biopsy was performed as previously described [21]. BALF cells, mucosal biopsies and PB were processed as previously described [21].

### Flow cytometry staining

BALF cells and PBMCs were stained for extra- and intracellular markers using the following antibodies: CD3-APC-eFluor780(SK7), CD4-AF700(OKT4), CD45RO-FITC(UCHl-1), CD95-APC(DX2), FoxP3-APC(PCH101) (eBiosciences) and CD25-PE(M-A251), CD25-PE-Cy7(M-A251), CD127-V450(hIL7R-M21) anti-CTLA-4-BV421(BNI3) (BD biosciences) and CD45RA-PE-Texas Red(MEM-56) (Invitrogen). Fixable Aqua Dead Cell Stain kit for 405 nm (Invitrogen, Molecular Probes) was used as live-dead marker. Cells were measured on a Flow cytometer LSRII (BD Biosciences).

### Suppression assays

Th cells and Tregs were sorted from PBMCs using a human CD4^+^CD25^+^Regulatory T Cell Isolation Kit (MACS, Miltenyi Biotec). Th cells were labeled with carboxyfluorescein succinimidyl ester (CFSE, Molecular Probes) according to manufacturer’s protocol. CFSE-labeled Th cells were stimulated with Anti-Biotin MACSiBead™ Particles (MACS, Miltenyi Biotec) and co-cultured with unlabeled Tregs. Th cell division was quantified based on serial halving of CFSE intensity, using algorithms provided by FlowJo software (Treestar).

Cytokine measurements in supernatants of co-cultures were performed using the Bio-Plex Pro Human Cytokine 17-plex Panel (Biorad), run on a Luminex 100 System (Luminex Corporation), according to manufacturer’s protocol.

### Survival and apoptosis assays

CD25^low-int^CD127^high^Th cells and CD25^int-high^CD127^low^Tregs were isolated from PBMCs using the BD FACSAria Cell Sorter (BD Biosciences) and cultured with 20 ng/ml recombinant human IL-2 (hIL-2; R&D systems). Survival was determined with DAPI Nucleic Acid Stain (Life Technologies)-negative cells. To measure Treg survival and apoptosis in co-culture with Th cells, CD4^+^ T cells were isolated from PBMCs using the human CD4^+^ T cell isolation kit (MACS, Miltenyi Biotec), cultured with 20 ng/ml hIL-2 and examined using the FITC Annexin V Apoptosis Detection Kit I (BD biosciences). To assess Treg apoptotic susceptibility, CD4^+^ T cells were cultured either with 20 ng/ml hIL-2 or with hIL-2 and 500 ng/ml soluble CD95L (recombinant human soluble FasL, Enzo Life Sciences).

### Statistical analyses

Comparisons were performed using a Mann–Whitney U test or Wilcoxon matched pairs test. Correlations were analyzed using Spearman’s rank-order correlation test. p-values were two sided, and analyses were performed using IBM SPSS Statistics 21. *p* < 0.05 was considered statistically significant.

## Results

### Increased proportions of circulating Tregs in patients developing chronic sarcoidosis

**Fig. 1:**
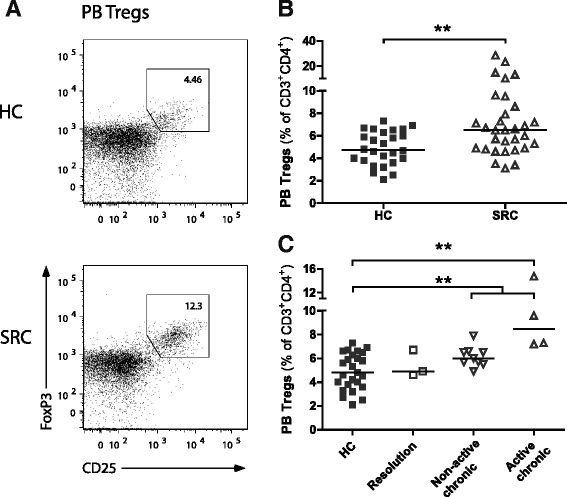
Increased proportions of circulating Tregs in patients developing chronic sarcoidosis. Treg proportions were determined in PB of HCs and SRC patients. **a** Representative flow cytometry analysis of an HC and SRC patient to determine Tregs in PB. **b** PB Treg proportions. **c** Subgroup analyses of PB Treg proportions at time of diagnosis in patients undergoing disease resolution, or developing (non-) active chronic disease. Statistics: *Horizontal lines* indicate the median and significance was determined using a Mann–Whitney U test, ** *p* < 0.01. *PB* peripheral blood, *HC* healthy control, *SRC* sarcoidosis

Conflicting results have been reported with regard to Treg proportions in lungs and PB of sarcoidosis patients [3]. Since a relative deficit of Tregs has been suggested to contribute to a persisting granulomatous response in chronic sarcoidosis [19, 22], we first quantified CD25^int-high^FoxP3^high^ Tregs in BALF and PB of active sarcoidosis patients and healthy controls by flow cytometry. No evidence was found for a relative deficit of Tregs in BALF of untreated patients with active sarcoidosis compared with healthy control BALF (Additional file 2: Figure S1), suggesting an intact migration of Tregs towards site of inflammation. Rather, in PB of these patients increased proportions of CD25^int-high^FoxP3^high^ Tregs were found (Fig. 1a,b). Interestingly, we determined the disease course of a subgroup of our study cohort with 2 years clinical follow-up, and found that in patients developing chronic sarcoidosis, but not in patients undergoing spontaneous resolution, significantly increased Treg proportions were detected at time of diagnosis, compared with healthy controls (Fig. 1c).

In summary, in untreated patients with active sarcoidosis, no evidence was found for a relative deficit of Tregs, neither systemically nor at the site of inflammation. In contrast, significantly increased proportions of circulating Tregs were observed, most prominently in patients developing chronic disease. These data show that Treg homeostasis is affected in active sarcoidosis, which could contribute to disease course.

### Circulating Tregs of sarcoidosis patients express adequate levels of FoxP3, CD25 and CTLA4

Since no numeral deficit of Tregs was found in patients at time of diagnosis, we questioned whether malfunctioning of circulating sarcoidosis Tregs is associated with an altered suppressive phenotype. Therefore, we analyzed circulating CD25^int-high^FoxP3^high^ Tregs of active sarcoidosis patients and healthy controls for expression of forkhead box P3 (FoxP3), CD25 and cytotoxic T lymphocyte antigen 4 (CTLA4) (three proteins pivotal for adequate Treg function [11]) by flow cytometry.

**Fig. 2:**
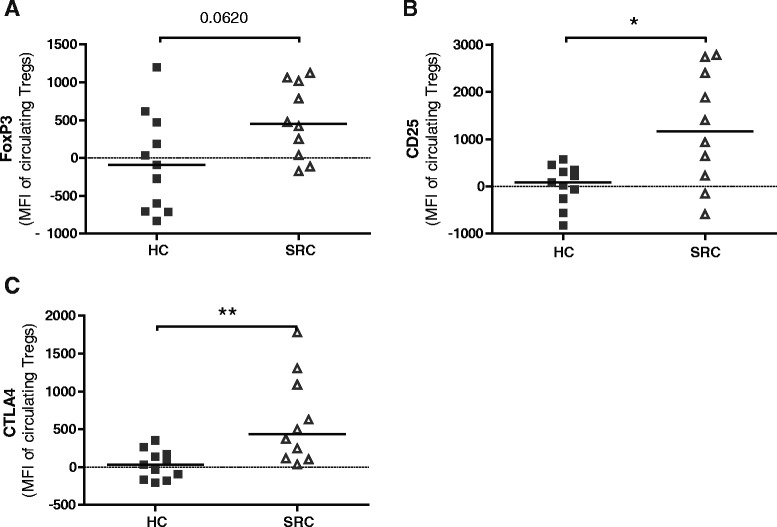
Adequate expression of FoxP3, CD25 and CTLA4 on sarcoidosis circulating Tregs. FoxP3, CD25 and CTLA4 expression was determined on circulating CD25^int-high^FoxP3^high^ Tregs of HC and SRC patients by flow cytometry. **a–c.** Mean fluorescence intensity of FoxP3 (**a**), CD25 (**b**) and CTLA4 (**c**). Mean fluorescence intensity was standardized to average expression in healthy control peripheral blood cells. Statistics: *Horizontal lines* indicate the median and significance was determined using a Mann–Whitney U test, * *p* < 0.05 ** *p* < 0.01. *FoxP3* forkhead box P3, *CTLA4* cytotoxic T lymphocyte antigen 4, *HC* healthy control, *SRC* sarcoidosis

We confirmed that PB-derived CD25^+^ Treg suppressive capacity on autologous Th proliferation and cytokine production was significantly less in sarcoidosis compared with healthy controls (Additional file 3: Figure S2). Circulating CD25^int-high^FoxP3^high^ Tregs of patients showed a trend towards increased expression levels of FoxP3 (Fig. 2a). Furthermore, CD25 and CTLA4 expression (downstream molecules of FoxP3) were significantly increased on PB CD25^int-high^FoxP3^high^ Tregs of sarcoidosis patients compared with healthy controls (Fig. 2b,c).

These data suggest that the impaired suppressive capacity of sarcoidosis PB-derived Tregs is not mediated by decreased expression of FoxP3 and its downstream molecules. Rather, a significant increase in CD25 and CTLA4 expression was found on circulating Tregs from sarcoidosis patients compared with healthy controls.

### Activated CD45RO^+^ Tregs from sarcoidosis patients highly express CD95

**Fig. 3:**
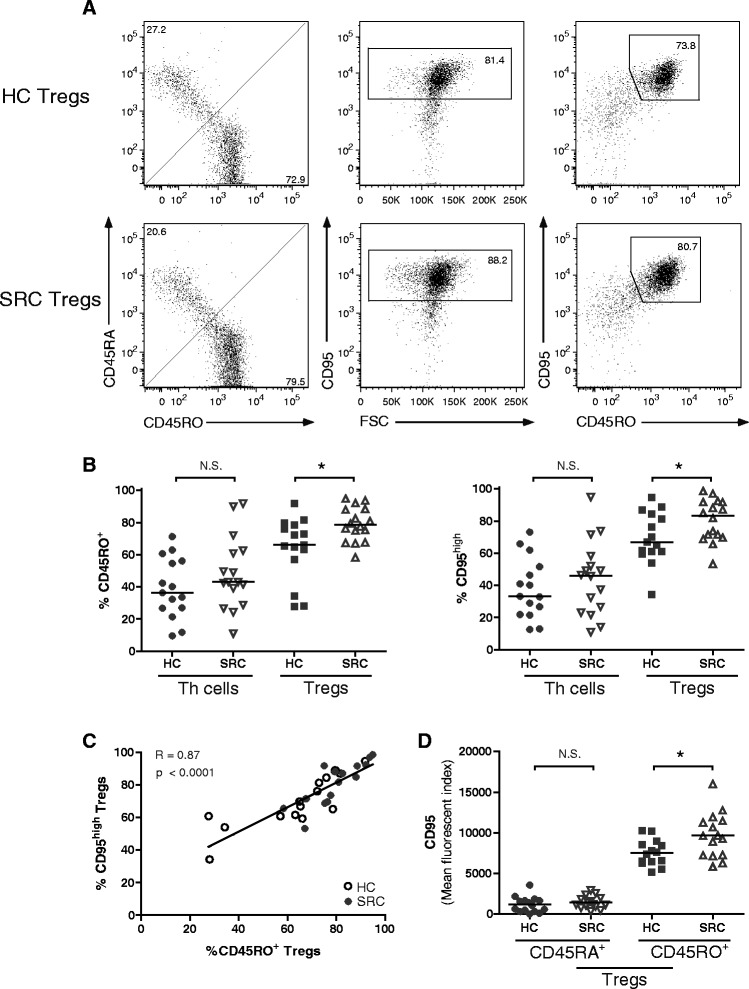
Activated CD45RO^+^ Tregs from sarcoidosis patients highly express CD95. The proportions of CD45RO and CD95 expressing Th cells and Tregs were determined in PB of HCs and SRC patients. **a** Representative flow cytometry analysis of an HC and SRC patient to determine the proportions CD45RO^+^, CD95^high^ and double positive Tregs (gated on CD3^+^CD4^+^CD25^int-high^CD127^low.^). **b** Proportions of CD45RO^+^ and CD95^high^ Th cells and Tregs. **c** Correlation between proportions CD45RO^+^ Tregs and CD95^high^ Tregs in PB. Open dots represent HC Tregs and closed dots represent SRC Tregs. **d** Mean fluorescence intensity of CD95 on CD45RA^+^ and CD45RO^+^ Tregs. Statistics: *Horizontal lines* indicate the median and significance was determined using a Mann–Whitney U test, * *p* < 0.05. Correlation was analysed using Spearman’s rank-order correlation test. Regression line with R and p-value are shown in the plot. *Th* T helper, *CD95* Fas; death receptor, *PB* peripheral blood, *HC* healthy control, *SRC* sarcoidosis

In healthy individuals, increased CD25 and CTLA4 expression on circulating Tregs has been associated with an activated, apoptotic-prone Treg population [20]. Therefore, increased CD25 and CTLA4 expression on sarcoidosis PB-derived Tregs could point towards increased apoptosis, counteracting their functionality. In order to analyze their apoptotic phenotype, CD45RO and CD95 (FAS; death receptor) expressing Th cells and Tregs were examined in PB of healthy controls and patients (Fig. 3a).

Within the PB Treg, but not Th cell population, proportions of CD45RO and CD95 expressing cells were significantly increased in sarcoidosis patients compared with healthy controls (Fig. 3b). The majority of CD45RO^+^ Tregs were CD95^+^ in both patients and controls (Fig. 3a) and their proportions strongly correlated (*p* < 0.0001, R = 0.87) (Fig. 3c). Importantly, surface expression of CD95 was significantly increased on sarcoidosis CD45RO^+^ Tregs compared with healthy control CD45RO^+^ Tregs, whereas CD95 expression on CD45RA^+^ Tregs was low and not different between healthy controls and patients (Fig. 3d).

Altogether, increased proportions of circulating, activated CD45RO^+^ Tregs were detected in sarcoidosis. Importantly, these activated CD45RO^+^ Tregs highly express CD95 in patients compared with controls, suggesting altered apoptotic susceptibility.

### Impaired survival of sarcoidosis Tregs

We questioned whether sarcoidosis-derived circulating Tregs would display altered survival. To investigate survival of sarcoidosis Th cells and Tregs, we isolated CD25^low-int^CD127^high^ Th cells and CD25^int-high^CD127^low^ Tregs from PB of patients and healthy controls. Proportions of CD25^int-high^FoxP3^high^ Tregs, as measured in the PB of our study subjects (Fig. 1), strongly correlated with proportions CD25^int-high^CD127^low^ Tregs (data not shown). These data underlined previously published data that CD4^+^CD25^int-high^CD127^low^ (sortable) can be used as surrogate marker for CD4^+^CD25^int-high^FoxP3^high^ Treg isolation and functional studies [23].

**Fig. 4:**
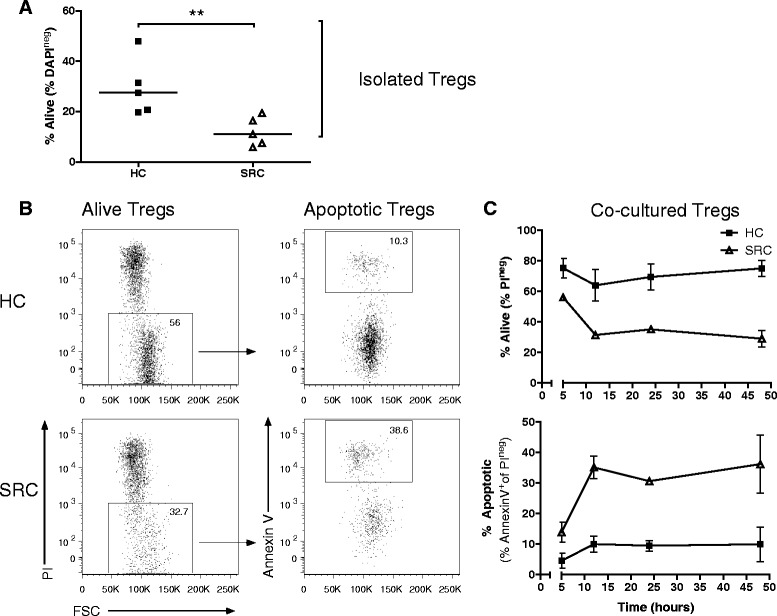
Impaired survival of sarcoidosis Tregs. Isolated Tregs (purity >97 %) were cultured with recombinant human IL-2. **a** Percentage alive Tregs at 72 hours of culture. *Horizontal line* indicates the median. Significance was determined using a Mann–Whitney U test, **p < 0.01. **b** Representative flow cytometry analysis of an HC and SRC patient after 12 hours co-culture with autologous Th cells to determine Treg survival and apoptosis. **c** Survival (*above*) and apoptosis (*below*) graph of Tregs cultured for 48 hours with autologous Th cells. *Dots* indicate mean +/− SEM of 3 HCs and 3 SRC patients. One representative experiment is shown of 3 independent experiments. *HC* healthy control, *SRC* sarcoidosis

Isolated cells were cultured with IL-2 and spontaneous survival was measured at 72 hours. Both healthy- and sarcoidosis-derived Tregs showed decreased survival compared with isolated Th cells (Additional file 4: Figure S3), confirming that Tregs are an apoptotic-prone population. Importantly, patient-derived Tregs showed significantly decreased survival compared with healthy control Tregs (Fig. 4a). This impaired survival was Treg-specific, since sarcoidosis-derived Th cells showed comparable survival to their healthy counterparts (Additional file 4: Figure S3). The survival defect of sarcoidosis Tregs was not restored when co-cultured with autologous Th cells (Fig. 4b and c(*upper plot*)). Moreover, increased proportions of apoptotic Tregs (annexin V^+^ and low FSC values) were observed in sarcoidosis (Fig. 4b and c(*lower plot*)).

These data provide evidence for an impaired survival of sarcoidosis Tregs, associated with increased apoptotic susceptibility.

### Increased sensitivity of sarcoidosis Tregs towards CD95L-mediated apoptosis

**Fig. 5:**
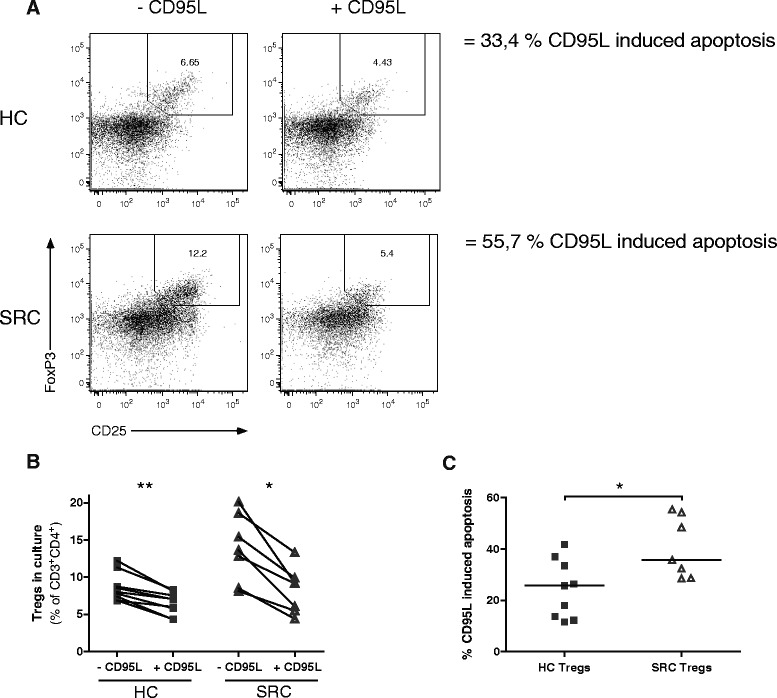
Increased sensitivity of sarcoidosis Tregs towards CD95L-mediated apoptosis. Freshly isolated Tregs were analysed for apoptotic susceptibility towards soluble CD95L. **a** Representative flow cytometry analysis of an HC and SRC patient after 20 hours of culture with IL-2 only or with IL-2 and soluble CD95L. Numbers indicate percentage of CD25^+^FoxP3^+^ Tregs in culture. Induced apoptosis by CD95L was calculated, using the following formula: ((% CD25^+^FoxP3^+^ Tregs cultured with IL-2 - % CD25^+^FoxP3^+^ Tregs cultured with IL-2 and CD95L)/(% CD25^+^FoxP3^+^ Tregs cultured with IL-2))*100 %. **b** Percentage Tregs in culture of 9 HCs and 7 SRC patients after 20 hours. Paired data is shown of the Treg cultures without or with soluble CD95L. **c** Percentage CD95L-induced apoptosis in HC and SRC Tregs. Statistics: *Horizontal lines* indicate the median. Significance was determined using a Mann–Whitney U test (**c**) or Wilcoxon matched pairs test (**b**), *p < 0.05 **p < 0.01. *CD95L* CD95 ligand, *HC* healthy control, *SRC* sarcoidosis

Since increased CD95 expression was observed on sarcoidosis CD45RO^+^ Tregs (Fig. 3) alongside impaired survival and increased apoptosis (Fig. 4), we investigated apoptotic susceptibility of freshly isolated CD4^+^ T cells towards soluble CD95L. Although both healthy control and sarcoidosis Tregs were sensitive towards CD95L-mediated apoptosis (Fig. 5a/b), CD95L-induced apoptosis was significantly enhanced in sarcoidosis Tregs compared with healthy controls (Fig. 5c).

These data provide evidence for increased susceptibility of sarcoidosis Tregs towards CD95L-mediated apoptosis.

## Discussion

In this study, for the first time, we provide evidence for an increased apoptotic susceptibility and impaired survival of sarcoidosis Tregs. Untreated patients with active pulmonary sarcoidosis showed enhanced CD95 expression levels on circulating activated CD45RO^+^ Tregs at time of diagnosis. Additionally, proportions of CD95^+^ and CD45RO^+^ circulating Treg were significantly increased. Furthermore, sarcoidosis Tregs were specifically more susceptible towards CD95L-induced apoptosis compared with healthy controls. Increased apoptosis likely contributes to the insufficient immunosuppressive function of sarcoidosis Tregs.

Failure of Treg-mediated immunosuppression is widely reported in autoimmune diseases [13] as well as granulomatous disorders, including AAV and HP [14, 15]. In sarcoidosis, impaired immunosuppression by Tregs has been found both in active and in chronic disease [4, 16–18]. It has been suggested to contribute to granuloma persistence [4] and was reported to recover during disease resolution [18]. Decreased immunosuppression by Tregs on Th cells may either result from deregulated Tregs (decreased proportions and/or malfunctioning), resistance of Th cells towards suppression or a combination of both [13]. We confirmed an impaired immunosuppressive function of PB-derived Tregs on Th cell proliferation and cytokine production. Although our assays cannot exclude that sarcoidosis Th cells contribute to this phenomenon, a previous study showed that sarcoidosis Th cells were responsive towards the suppressive capacity of healthy Tregs [18]. Furthermore, we did not find evidence for a numeral deficit of Tregs, neither systemically nor in the lungs of untreated patients with active pulmonary sarcoidosis at time of diagnosis. Rather, we and others [4, 18] found increased proportions of circulating Tregs in active sarcoidosis patients.

Importantly, we are the first to report that increased proportions of circulating Tregs at time of diagnosis are mainly attributable to patients who develop chronic disease and not patients who undergo spontaneous resolution. Interestingly, *Prasse et al.* previously found significantly decreased Treg proportions in lungs of patients who develop chronic active disease compared with controls, but not in patients who undergo spontaneous resolution [19]. Taken together, these data suggest that enhanced circulating Treg proportions in sarcoidosis reflect impaired migration towards the site of inflammation. Alternatively, during homeostatic conditions Tregs rapidly adjust their numbers in response to IL-2 variations, directly reflecting Th cell activity. Therefore, in agreement with published findings [12], it is also conceivable that at time of diagnosis in sarcoidosis patients circulating Treg proportions expand as a result of local Treg failure, specifically in patients who will develop chronic sarcoidosis. Therefore, the numbers of circulating Tregs at the time of diagnosis potentially serve as a new biomarker indicating need for immunosuppressive drugs that restore immune homeostasis. To further address this issue prospective studies are warranted.

To the best of our knowledge, it thus far remained unclear what mechanism(s) underlies Treg dysfunction in PB of active sarcoidosis patients. We did not find differences in FoxP3 expression or diminished expression of its downstream molecules CD25 or CTLA4 in circulating Tregs of sarcoidosis patients. Interestingly, we found that Tregs in lymph nodes from sarcoidosis patients have reduced CTLA4 expression compared with controls (Broos et al., AJRCCM, *in press*), indicating that CTLA4 expression levels are differentially regulated between compartments. Thus, despite adequate Treg proportions and expression of FoxP3-downstream effector molecules, sarcoidosis-derived PB Tregs fail to suppress autologous Th cell responses.

Pro- and anti-apoptotic pathways govern Treg homeostasis [12]. In this study a Treg-specific survival defect was found in sarcoidosis patients, whereby CD95L-mediated apoptosis was increased. Although factors determining T cell sensitivity towards CD95L-mediated apoptosis remain to be fully elucidated, T cell activation status and CD95 expression appear critical [24]. Indeed, our data argue that in sarcoidosis circulating activated CD45RO^+^ Tregs highly expressing CD95 compared with control CD45RO^+^ Tregs, can contribute to impaired Treg survival.

Our finding that in sarcoidosis Tregs are hypersensitive towards CD95L-mediated apoptosis parallels earlier findings in SLE [25] and adds to our previously described similarities between sarcoidosis and systemic autoimmune disorders, which include the involvement of pathogenic IFN-γ-producing Th17 cells [26, 27]. In SLE increased sensitivity of Tregs towards CD95L-mediated cell death was proposed to exacerbate the extent of tissue damage during flares [25]. Increased Treg apoptosis in sarcoidosis might hamper restoration of the immune balance and contribute to the development of (chronic) sarcoidosis. Interestingly, CD95 signaling has previously been suggested to contribute to chronic sarcoidosis pathology, since an activating CD95 promotor polymorphism (−670A) has been associated with disease severity in Afro-American patients [28]. This -670A variant in the CD95 gene promotor has also been associated with SLE and was shown to influence CD95 gene expression, whereby the A (instead of the G) allele induces increased CD95 transcription [29]. It is tempting to speculate that increased transcription of CD95 in sarcoidosis, mainly affecting activated CD45RO^+^ Tregs due to their physiological CD95 expression [24], contributes to the development of chronic sarcoidosis. Importantly, increased Treg sensitivity towards CD95L-mediated apoptosis does not seem to be a general consequence of chronic inflammation, since it was excluded in multiple sclerosis and granulomatosis with polyangiitis [30, 31].

Previously it has been suggested that decreased apoptosis of antigen-specific T cells might contribute to granuloma persistence in sarcoidosis [22]. However, sarcoidosis-derived BALF and PB T cells highly express CD95 [32, 33] and show signs of apoptosis [34]. Lymphocytes infiltrating and surrounding sarcoid granulomas express CD95L [34], and increased amounts of soluble CD95L are found in BALF and serum of patients [35]. Most interesting, patients with chronic sarcoidosis and need for therapy show an increased expression of CD95 on BALF and PB T cells (including CD45RO on PB T cells) at time of diagnosis compared with patients undergoing spontaneous resolution of disease [33]. These data imply that increased apoptosis of T cells contributes to ongoing inflammation. However, the role of CD95L-mediated apoptosis in the homeostasis of pro- and anti-inflammatory T cell subsets (i.e. Th cells versus Tregs, respectively) in sarcoidosis thus far remained unclear [34]. In this study, for the first time, evidence is provided for a Treg specific survival defect, which can lead to an imbalance between pro-inflammatory Th cells and properly functioning anti-inflammatory Tregs, resulting in on going inflammation.

The anti-TNF agent infliximab is known to induce Treg functionality in RA [7]. Induction of Treg survival may very well contribute to its therapeutic effect as observed in patients with chronic sarcoidosis [5]. Investigation of Treg proportions present in or around the granulomas, their functional capacities, and apoptosis susceptibility during the natural course of disease and in response to therapy should further unravel the role of Tregs in the development of chronic sarcoidosis. Research into this field will help determine whether improvement of Treg survival, e.g. by other immunosuppressive drugs, such as rapamycin [36, 37], holds a promising new therapeutic approach for chronic sarcoidosis patients.

## Conclusion

In conclusion, this study is the first to demonstrate a role for deregulated Treg survival, mediated by CD95-signaling, in untreated patients with active pulmonary sarcoidosis. Increased apoptosis likely contributes to the insufficient immunosuppressive function of sarcoidosis Tregs. Further research into this field will help determine whether improvement of Treg survival holds a promising new therapeutic approach for (chronic) sarcoidosis patients.

## Additional files

Additional file 1: Table S1. Study subject characteristic. *Median (minimum-maximum). † Anonymous blood donors. ‡ Two of these patients had Stage I and two had Stage II sarcoidosis, determined by CT scan. § Disease course of a subgroup of patients (*n* = 28) was determined 2 years after study inclusion. Resolution of disease was defined by the absence of abnormalities on the chest X-ray and clinical symptoms (*n* = 5). Patients with residual abnormalities on chest X-ray, but without need for treatment were designated as non-active chronic (*n* = 15); and patients with need for treatment (*n* = 8) as active chronic (*Prasse et al., AJRCCM (2010) 182(4):540–8*). (DOCX 21 kb) Additional file 2: Figure S1. Treg proportions were determined in BALF of HCs and SRC patients. **A.** Representative flow cytometric analysis of an HC and SRC patient to determine Treg proportions in BALF. **B.** BALF Treg proportions. *Statistics:* Horizontal lines indicate the median and significance was determined using a Mann–Whitney U test. *Abbreviations:* BALF: broncho-alveolar lavage fluid, HC: healthy control, SRC: sarcoidosis. (PDF 386 kb) Additional file 3: Figure S2. Freshly isolated Th cells were CFSE-labelled, TCR-stimulated and cultured without or with autologous Tregs. **A.** Flow cytometry analysis of one HC and SRC patient after 4 days of culture. **B.** Percentage suppression by Tregs on autologous Th cell proliferation. Percentage suppression was calculated as follows: ((%divided Th cells only - % divided Th cells co-cultured with Tregs)/(%divided Th cells only))*100 %. Horizontal lines indicate the median and significance was determined using a Mann–Whitney U test, **p* < 0.05. **C-D.** Amount of IL-2 (**C**) or IFN-γ (**D**) measured in culture supernatant of suppression assays at day 4. Paired data is shown of Th cell cultures without or with autologous Tregs. Significance was determined using a Wilcoxon matched pairs test, **p* < 0.05. *Abbreviations:* Th: T helper, CFSE: carboxyfluorescein succinimidyl ester, TCR: T cell receptor, HC: healthy control, SRC: sarcoidosis, NS = not significant. (PDF 550 kb) Additional file 4: Figure S3. Isolated Th cells and Tregs were cultured with recombinant human IL-2. **A.** Percentage alive Th cells and Tregs at 72 hours of culture is shown. Horizontal line indicates the median. Significance was determined using a Mann–Whitney U test, **p* < 0.05 ***p* < 0.01. *Abbreviations:* Th: T helper, HC: healthy control, SRC: sarcoidosis, NS: not significant. (PDF 329 kb)
