# Supplementary material for: Impaired survival of regulatory T cells in pulmonary sarcoidosis
Source: Respir Res. 2015 Sep 16;16(1):108. doi: 10.1186/s12931-015-0265-8 (PMC4574219; doi:10.1186/s12931-015-0265-8)
Supplement: Additional file 1: Table S1. — Study subject characteristic. *Median (minimum-maximum). † Anonymous blood donors. ‡ Two of these patients had Stage I and two had Stage II sarcoidosis, determined by CT scan. § Disease course of a subgroup of patients (n = 28) was determined 2 years after study inclusion. Resolution of disease was defined by the absence of abnormalities on the chest X-ray and clinical symptoms (n = 5). Patients with residual abnormalities on chest X-ray, but without need for treatment were designated as non-active chronic (n = 15); and patients with need for treatment (n = 8) as active chronic (Prasse et al., AJRCCM (2010) 182(4):540–8). (DOCX 21 kb) [file 12931_2015_265_MOESM1_ESM.docx]

| Subject Characteristics | Healthy controls (n=47) | Sarcoidosis (n=58) |
| --- | --- | --- |
| Age (y)* | 36 (19-59) | 44 (17-81) |
| Sex (Male/Female/Unknown†) | 23/22/2 | 31/27/0 |
| Ethnicity (n (%)) |  |  |
| White | 34 (72,3) | 35 (60,3) |
| Black | 2 (4,3) | 12 (20,7) |
| Asian | 2 (4,3) | 6 (10,3) |
| Hispanic | 0 (0) | 1 (1,7) |
| Unknown† | 9 (19,1) | 4 (6,9) |
| Scadding Stage (n (%)) |  |  |
| Stage 0 |  | 5 (8,6) |
| Stage I |  | 25 (43,1) |
| Stage II |  | 21 (36,2) |
| Stage III |  | 3 (5,2) |
| Stage IV |  | 0 (0,0) |
| Unknown |  | 4 (6,9)‡ |
| CD4/8 ratio BALF (n=46)* |  | 4,3 (0,5 – (>)10) |
| BALF lymphocytes (n=46)* |  | 25,0 (0 - 84) |
| Diagnosis was assessed by (n (%)) |  |  |
| Trans- and/or endobronchial biopsy |  | 18 (31,0) |
| E(B)US-FNA |  | 17 (29,3) |
| BALF alveolitis + CD4/CD8 ratio > 3.5 |  | 22 (38,0) |
| Mediastinoscopy |  | 1 (1,7) |
| Disease course^§^ (n (%)) |  |  |
| Resolution |  | 5 (8,6) |
| Non-active chronic |  | 15 (25,9) |
| Active chronic |  | 8 (13,8) |
| Lost to follow up |  | 12 (20,7) |
| Not yet determined |  | 18 (31,0) |

**Table S1. Study subject characteristics**
