# Supplementary figures and images for: Impaired survival of regulatory T cells in pulmonary sarcoidosis
Source: Respir Res. 2015 Sep 16;16(1):108. doi: 10.1186/s12931-015-0265-8 (PMC4574219; doi:10.1186/s12931-015-0265-8)

**A.** BALF Tregs

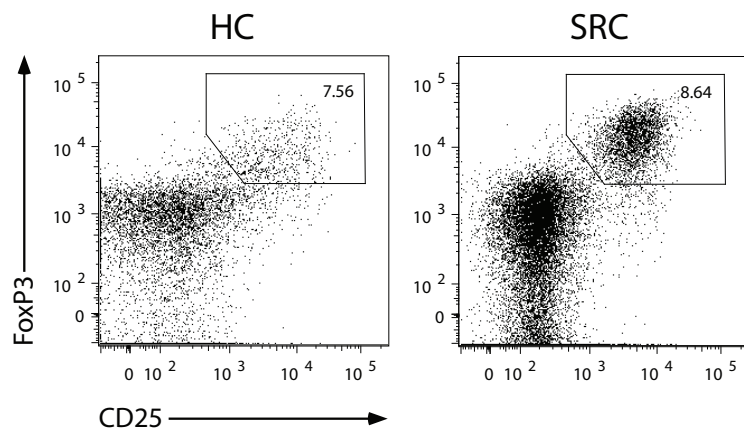

**B.**

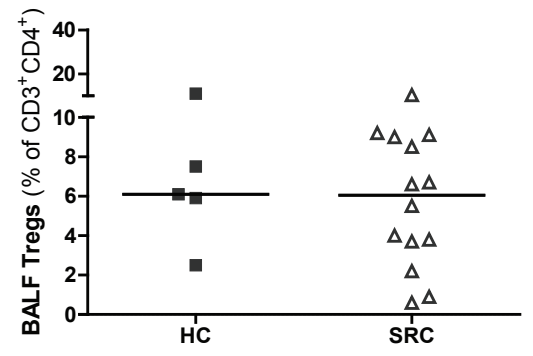

**Figure S1.**

Supplement: Additional file 2: Figure S1. — Treg proportions were determined in BALF of HCs and SRC patients. A. Representative flow cytometric analysis of an HC and SRC patient to determine Treg proportions in BALF. B. BALF Treg proportions. Statistics: Horizontal lines indicate the median and significance was determined using a Mann–Whitney U test. Abbreviations: BALF: broncho-alveolar lavage fluid, HC: healthy control, SRC: sarcoidosis. (PDF 386 kb) [file 12931_2015_265_MOESM2_ESM.pdf]

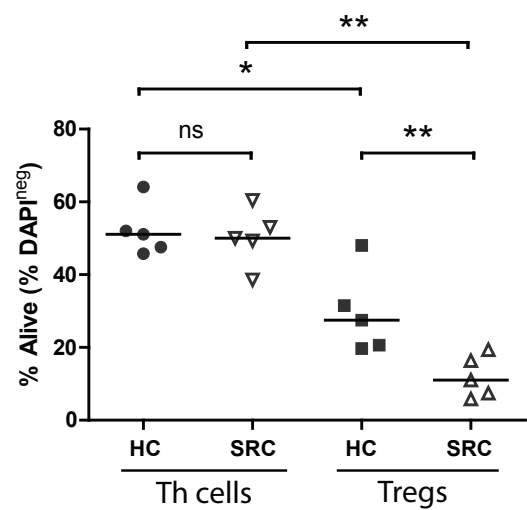

**Figure S3.**

Supplement: Additional file 4: Figure S3. — Isolated Th cells and Tregs were cultured with recombinant human IL-2. A. Percentage alive Th cells and Tregs at 72 hours of culture is shown. Horizontal line indicates the median. Significance was determined using a Mann–Whitney U test, *p < 0.05 **p < 0.01. Abbreviations: Th: T helper, HC: healthy control, SRC: sarcoidosis, NS: not significant. (PDF 329 kb) [file 12931_2015_265_MOESM4_ESM.pdf]
