# Supplementary material for: Impaired survival of regulatory T cells in pulmonary sarcoidosis
Source: Respir Res. 2015 Sep 16;16(1):108. doi: 10.1186/s12931-015-0265-8 (PMC4574219; doi:10.1186/s12931-015-0265-8)
Supplement: Additional file 3: Figure S2. — Freshly isolated Th cells were CFSE-labelled, TCR-stimulated and cultured without or with autologous Tregs. A. Flow cytometry analysis of one HC and SRC patient after 4 days of culture. B. Percentage suppression by Tregs on autologous Th cell proliferation. Percentage suppression was calculated as follows: ((%divided Th cells only - % divided Th cells co-cultured with Tregs)/(%divided Th cells only))*100 %. Horizontal lines indicate the median and significance was determined using a Mann–Whitney U test, *p < 0.05. C-D. Amount of IL-2 (C) or IFN-γ (D) measured in culture supernatant of suppression assays at day 4. Paired data is shown of Th cell cultures without or with autologous Tregs. Significance was determined using a Wilcoxon matched pairs test, *p < 0.05. Abbreviations: Th: T helper, CFSE: carboxyfluorescein succinimidyl ester, TCR: T cell receptor, HC: healthy control, SRC: sarcoidosis, NS = not significant. (PDF 550 kb) [file 12931_2015_265_MOESM3_ESM.pdf]

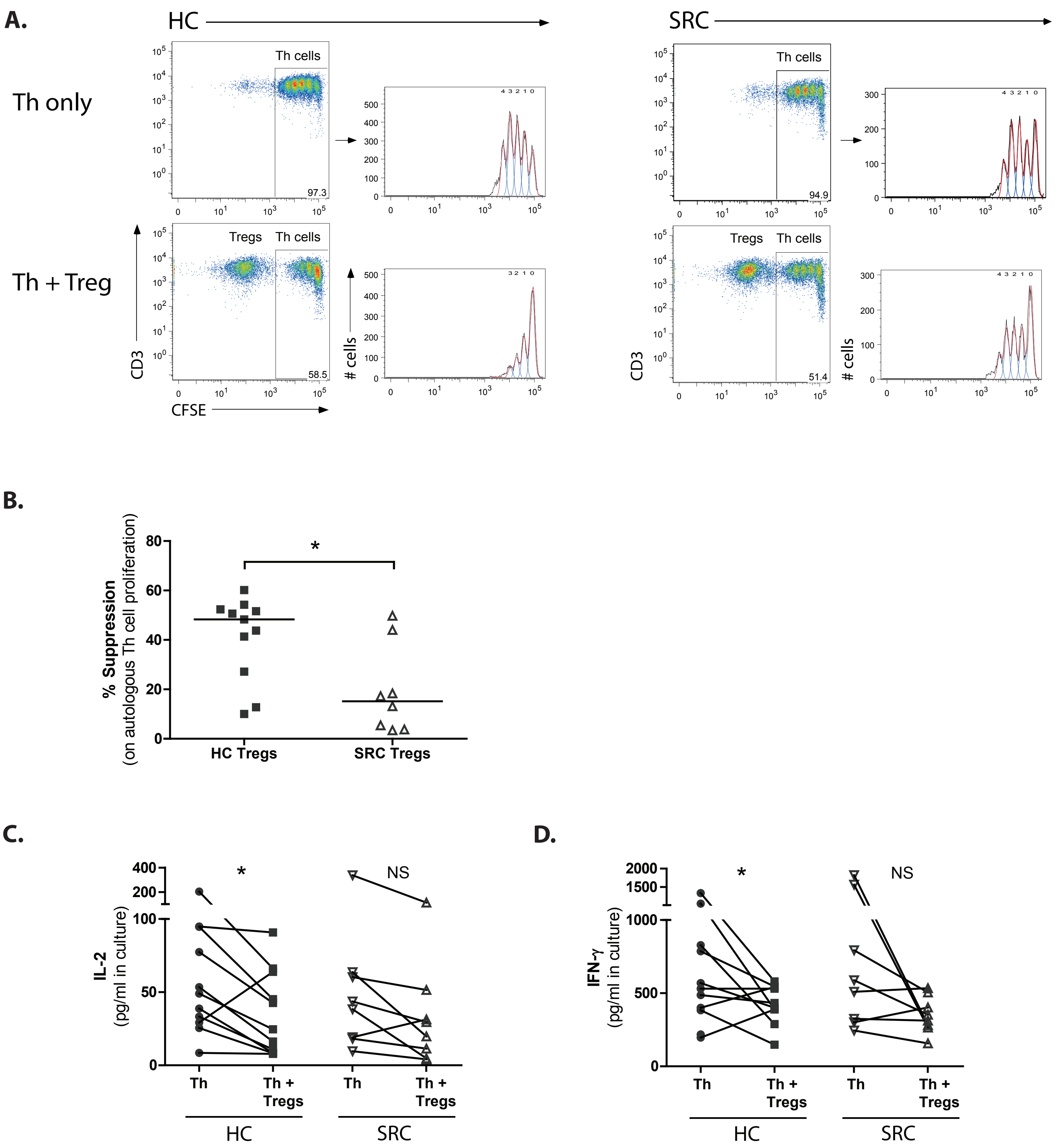
A.
HC
SRC
Th only
Th + Treg
Th cells
Tregs
97.3
58.5
94.9
51.4
CD3
CFSE
cells
B.
% Suppression
(on autologous Th cell proliferation)
HC Tregs
SRC Tregs
*
C.
IL-2
(pg/ml in culture)
D.
IFN-γ
(pg/ml in culture)
Th
Th + Tregs
HC
SRC
NS

Figure S2.
